# Genetic analysis using next-generation sequencing and multiplex ligation probe amplification in Chinese aniridia patients

**DOI:** 10.1186/s13023-024-03388-3

**Published:** 2024-10-24

**Authors:** Li Wang, Qingdan Xu, Wentao Wang, Xinghuai Sun, Yuhong Chen

**Affiliations:** 1 Department of Ophthalmology and Visual Science, Eye and ENT Hospital, Shanghai Medical College, Fudan University, 83 Fenyang Road, Shanghai, 200031 China; 2 Center Laboratory, Eye and ENT Hospital, Shanghai Medical College, Fudan University, Shanghai, China; 3 NHC Key Laboratory of Myopia and Related Eye Diseases, Chinese Academy of Medical Sciences, Shanghai Key Laboratory of Visual Impairment and Restoration (Fudan University), Shanghai, China; 4 State Key Laboratory of Medical Neurobiology and MOE Frontiers Center for Brain Science, Institutes of Brain Science, Fudan University, Shanghai, China

**Keywords:** Congenital aniridia, Panel-based next-generation sequencing, Multiplex ligation probe amplification, *PAX6*, *FOXC1*, *BCOR*

## Abstract

**Background:**

Congenital aniridia is a rare pan-ocular disease characterized by complete irideremia, partial iridocoloboma. The progressive nature of aniridia is frequently accompanied by secondary ocular complications such as glaucoma and aniridia-associated keratopathy, which can lead to severely impaired vision or blindness. The genetic basis of aniridia has been the subject of numerous studies, leading to the development of innovative therapeutic options based on *PAX6* nonsense mutations. Specific knowledge of the genetics of aniridia has become increasingly important. To report the clinical features, elucidate the genetic etiology, and reveal the mutational spectrum of congenital aniridia in the Chinese population, sixty patients with congenital aniridia from 51 families were recruited. Candidate genes associated with developmental eye diseases were identified and analyzed using panel-based next-generation sequencing (NGS), and mutations were confirmed through polymerase chain reaction and Sanger sequencing. Multiplex ligation probe amplification (MLPA) of *PAX6* and *FOXC1* was performed to detect copy number variations in the patients without intragenic mutations.

**Results:**

Clinical examination revealed complete iris hypoplasia in 58 patients and partial iris hypoplasia in two patients. Additionally, two patients were diagnosed with Wilms’ tumor-aniridia-genital anomalies-retardation syndrome and nephroblastoma. By combining panel-based NGS and MLPA, 43 intragenic mutations or deletions of *PAX6*, *FOXC1*, and *BCOR* were identified in 59 patients, including 33 point mutations (76.7%) in 43 patients and 10 deletions (23.3%) in 16 patients. The total detection rate was 98.3%. Phenotypic variation was observed between and within families.

**Conclusions:**

Variations in *PAX6* and its adjacent regions were the predominant causes of aniridia in China. In addition to intragenic point mutations in *PAX6*, deletion of *PAX6* or its adjacent genes is a common cause of congenital aniridia. Furthermore, *FOXC1* is an important gene associated with congenital aniridia. The combination of panel-based NGS and MLPA significantly enhanced the detection rate of gene mutations in patients with congenital aniridia.

**Supplementary Information:**

The online version contains supplementary material available at 10.1186/s13023-024-03388-3.

## Background

Congenital aniridia is a rare pan-ocular disease characterized by complete irideremia, partial iridocoloboma, aniridia-associated keratopathy, glaucoma, lens opacity or dislocation, macular dysplasia, optic nerve dysplasia, and nystagmus. In addition, the disease can also manifest as systemic abnormalities, such as hyposmia, neurologic abnormalities, diabetes, obesity, renal neoplasms, and genitourinary anomalies, collectively known as aniridia syndrome, including Wilms’ tumor-aniridia-genital anomalies-retardation (WAGR) syndrome [11], WAGRO syndrome [16], and Gillespie syndrome (non-progressive cerebellar ataxia, intellectual disability, and iris hypoplasia) [27].

The *PAX6* (paired box gene 6; OMIM # 607108), which is located on chromosome 11p13, encodes a highly conserved transcription factor that contains two DNA-binding domains (a paired domain and a homeodomain linked by a linker region) and a proline-serine-threonine-rich transactivation domain [12]. Heterozygous mutations or deletions within *PAX6* or adjacent regions are the major causes of aniridia, which can disrupt normal development of the eye [28]. The inheritance is predominantly autosomal dominant, with high penetrance but variable expressivity. Other genes, including *FOXC1*, *CYP1B1*, *PITX2* and *FOXE3*, have been identified as potential contributors to iris hypoplasia and aniridia-like phenotypes [20, 22, 23, 25, 26, 39].

The progressive nature of aniridia is frequently accompanied by secondary ocular complications such as glaucoma and aniridia-associated keratopathy, which can lead to severely impaired vision or blindness. Over the past few decades, the genetic basis of aniridia has been the subject of numerous studies, leading to the development of innovative therapeutic options based on *PAX6* nonsense mutations. One such option is nonsense suppression therapy, which utilizes small molecular compounds to suppress translation termination at in-frame premature termination codons, allowing translation elongation of the mRNA to continue in the original ribosomal reading frame, thus generating a full-length polypeptide [14, 43, 44]. Therefore, specific knowledge of the genetics of aniridia has become increasingly important. The present study investigated 60 Chinese patients with congenital aniridia and their family members to elucidate the mutational spectrum.

## Methods

### Patients and sample collection

Patients were consecutively enrolled between January 2017 and December 2021 at the Eye, Ear, Nose, and Throat Hospital (EENT Hospital), Shanghai Medical College, Fudan University, Shanghai, China. The study adhered to the tenets of the Declaration of Helsinki and was approved by the Institutional Review Board of the EENT Hospital. Informed consent, including consent for publication, was obtained from each participant and/or their legal guardians after a full explanation of the purpose, procedures, and possible consequences of the study prior to the procedures. We enrolled 51 probands with congenital aniridia and their family members. Of these, 10 probands had a family history, whereas the remaining 41 probands were sporadic. Patients who were able to cooperate underwent standard ophthalmological examination, including best-corrected visual acuity (BCVA, recorded in logMAR visual acuity), slit-lamp biomicroscopy, fundus examination, intraocular pressure measurement (IOP), ultrasound biomicroscopy, and optical coherence tomography (OCT). Children under 3 years of age with glaucoma underwent IOP and ultrasound biomicroscopy (UBM) examinations under general anesthesia prior to glaucoma surgery. Sixteen patients underwent UBM (MD-300 L; MEDA Co., Ltd. Tianjin, China) to assess anterior chamber angle. For macular fovea assessment, 20 patients were examined through ophthalmoscopy, and 15 patients underwent OCT (Software Version: 6.12.4, Heidelberg Engineering GmbH, or Cirrus OCT 5000, SW Ver: 7.0.1.290, Carl Zeiss Meditec, Inc.). OCT structural grading of foveal hypoplasia was based on the Leicester grading system for foveal hypoplasia [37]. Genomic DNA samples were extracted from peripheral blood of all participants using the QIAamp^®^ DNA Mini Kit (Qiagen, Hilden, Germany). Quantity and purity of gDNA were assessed employing Qubit^®^ 3.0 Fluorometer (Invitrogen, Carlsbad, CA, USA) and NanoDrop-One (Thermo Scientific, Wilmington, DE, USA).

### Targeted enrichment panel

Genomic testing was performed using panel-based next-generation sequencing (NGS) established by Amplicon Gene (Shanghai, China). It was custom-designed to include the exon sequences of 289 genes involved in ocular anterior segment dysgenesis, with an extension of the exon area by 50 bp on either side (See Supplement 2 for a summary of the 289 genes). Sequencing libraries were quality controlled and quantified using Agilent 2100 Bioanalyzer (Agilent Technologies) and Qubit^®^ 3.0 Fluorometer (Invitrogen). Sequencing of the libraries was performed on an Illumina Novaseq600 platform (Illumina Inc, San Diego, CA, USA) in high output mode, 2 × 150 cycles, with TruSeq SBS chemistry.

### Variant analysis

After filtering low-quality reads using cutadapt (v2.6; https://cutadapt.readthedocs.io/en/stable/), raw sequencing reads were mapped to the human reference genome (hg19) with BWA (version 0.7.12-r1039), and post-processing was performed according to Genome Analysis Toolkit version 3.7 (GATK) best practice. Variant calling was performed utilizing the Haplotypecaller/GATK v3.7, followed by quality control and variant filtering. Duplicates were marked using the MarkDuplicates/GATK v3.7. The variants were annotated employing the ANNOVAR software. The variants in the intronic, upstream, and downstream sites were removed, and those in the exonic and splicing functional regions were retained. The variants were not considered if they had a read depth < 10×, variant allele frequency < 0.2, a minor allele frequency (MAF) > 0.01 in databases including 1000 Genome project, Genome Aggregation Database, and Exome Aggregation Consortium. The novel variants were classified as being pathogenic, likely pathogenic, or of uncertain clinical significance according to the interpretation principles of the HGMD sequence variants and the American College of Medical Genetics (ACMG) guidelines. To verify the variants, Sanger sequencing was conducted on probands and other family members, if available, as previously described. The sequencing results were compared with the published cDNA sequences for *PAX6* (GenBank NM_000280.5) and *BCOR* (GenBank NM_001123385.2). Variations were described using HGVS nomenclature [9].

### Multiplex ligation-dependent probe amplification

For patients who did not have intragenic mutations in *PAX6*, *FOXC1*, or other reported candidate genes based on panel-based exome sequencing analysis, multiplex ligation probe amplification (MLPA) was performed to detect large deletions or duplications in these genes according to the manufacturer’s instructions: SALSA MLPA Kit P219-B3 for *PAX6*, and SALSA MLPA Probemix P267 Dandy-Walker for *FOXC1* (MRC Holland, Amsterdam, Netherlands). The results were analyzed using Genemapper4.0. Copy number variations were identified utilizing Coffalyser. Net (MRC, Holland). A peak area between 0.8 and 1.2 times was considered normal; however, peak areas below 0.65 represent deletions and those above 1.3 represent duplications.

## Results

### Clinical characteristics

This study enrolled 60 patients from 51 unrelated families (41 sporadic and 10 with family histories). The age at first examination ranged from 4 months to 80 years, and the BCVA (recorded in logMAR visual acuity) varied from 0.3 to no light perception. The clinical features of the patients are presented in Table 1.

**Table 1:** Clinical findings for 60 patients in this study

Patient No.	Inheritence	Gender	Age	Clinical diagnosis	Foveal hypoplasia/Grading based on OCT**	Other features	BCVA (LogMAR)(OD/OS)	IOP (mmHg)	Axial length (mm)
1	S	female	34ys	congenital aniridia	yes	cataracts	2.9/2.2	ND	22.76/22.01
2	S	male	11ys	congenital aniridia	yes	cataracts	ND	ND	20.92/19.61
3	F	female	15ys	congenital aniridia	yes	cataracts, nystagmus, lens subluxation	2.9/2.2	19/20	19.33/19.51
3-2	F, mother of No.3	female	42ys	congenital aniridia	yes	cataracts	ND	ND	ND
4	F	female	23ys	congenital aniridia	yes	cataracts, glaucoma	2.7/-2.903	44/46	24.80/-
4-2	F, father of No.4	male	48ys	congenital iridocoloboma	yes	cataracts, hyperpresbyopia	ND	16/14	23.67/25.35
5	S	male	15ys	congenital aniridia	yes	glaucoma	1.3/1.3	46/35	30.45/27.91
6	S	male	10ys	congenital aniridia	yes	cataracts	ND	12/14	ND
7	S	female	6ys	congenital aniridia	ND	nystagmus	ND	14/14	ND
8	S	male	38ys	congenital aniridia	yes	cataracts, glaucoma	-3.505/1.0	43/16	28.96/26.58
9	S	male	4ys	congenital aniridia	yes	glaucoma, nephroblastoma	ND	37/41	28.95/24.78
10	S	male	22ys	congenital aniridia	yes/Grade 4/4	cataracts, glaucoma, nystagmus	1.1/1.0	47/36	24.36/23.62
11	S	male	8ys	congenital aniridia	ND	cataracts, glaucoma	ND	16/27	25.16/25.87
12	S	female	2ys	congenital aniridia	ND	glaucoma, nystagmus	ND	29/35	22.72/21.59
13	S	male	6mos	congenital aniridia	ND	glaucoma, nystagmus	ND	16/27	20.12/21.24
14	S	male	2ys	congenital aniridia	ND	nystagmus	ND	ND	ND
15	S	male	12ys	congenital aniridia	yes	glaucoma, nystagmus	1.2/1.7	26/12	23.33/24.08
16	S	male	1ys	congenital aniridia	ND	glaucoma, nystagmus	ND	30.4/30	24.38/26.56
17	S	male	2ys	congenital aniridia	yes	glaucoma, nystagmus	ND	21/35	20.60/35.80
18	S	male	6ys	congenital aniridia	yes/Grade 4/4	cataracts, hyperpresbyopia	1.3/1.3	11/11	22.89/22.71
19	S	male	8ys	congenital aniridia	yes/Grade 4/ND	cataracts, glaucoma	ND	26/32	26.56/25.81
20*	F	female	27ys	congenital aniridia	yes/Grade 3/4	cataracts, glaucoma	0.6/1.3	45.5/46	23.50/24.78
21	F	male	8ys	congenital aniridia	yes	cataracts, glaucoma	1.0/2.0	18/38	ND
21-2	F, father of No.21	male	43ys	congenital aniridia	yes	cataracts	ND	15.6/16.6	ND
22	S	male	3ys	congenital aniridia	yes/Grade 4/4	cataracts, hyperpresbyopia	ND	12.9/15.3	ND
23	F	male	7ys	congenital aniridia	yes/Grade 4/4	cataracts	1.05/1.05	18.0/18.5	ND
23-2	F, mother of No.23	female	27ys	congenital aniridia	yes/Grade 3/3	cataracts	0.7/0.8	21.9/16.1	ND
24	S	male	12ys	congenital aniridia	yes	cataracts, glaucoma, left eye lens subluxation	ND	13/24	ND
25	S	female	32ys	congenital aniridia	yes	cataracts	ND	ND	ND
26	S	male	10mos	congenital aniridia	ND	ND	ND	ND	18.3/18.0
27	F	male	18ys	congenital aniridia	ND	cataracts	ND	ND	ND
27-2	F, mother of No.27	female	40ys	congenital aniridia	yes/Grade 4/3	cataracts, glaucoma	-3.505/2.9	48.6/21.8	ND
28	S	female	2ys	congenital aniridia	yes	nystagmus	ND	ND	19.05/19.59
29	F	male	25ys	congenital aniridia	yes/Grade ND/4	cataracts, glaucoma, nystagmus	-3.505/1.0	ND	-/25.63
29-2	F, father of No.29	male	49ys	congenital aniridia	yes	cataracts	1.4/1.0	29.6/26.8	ND
30	S	female	5ys	congenital iridocoloboma	yes/Grade 3/3	nystagmus	1.0/1.0	13.3/15.8	ND
31	S	male	2ys	congenital aniridia	ND	cataracts, nephroblastoma	ND	ND	ND
32	S	male	5mos	congenital aniridia	ND	cataracts	ND	25/24	ND
33	S	male	2ys	congenital aniridia	ND	ND	ND	ND	ND
34	S	male	4mos	congenital aniridia	ND	cataracts	ND	ND	ND
35	S	female	4mos	congenital aniridia	ND	left external ear dysplasia	ND	ND	ND
36	S	male	1ys	congenital aniridia	ND	glaucoma	ND	31/32	21.20/22.86
37	S	female	1ys	congenital aniridia	ND	nystagmus	ND	ND	ND
38	S	male	4mos	congenital aniridia	ND	ND	ND	15/12	20.8/21.1
39	S	female	5mos	congenital aniridia	ND	nystagmus	ND	17.9/18.4	20.14/20.54
40	S	female	10ys	congenital aniridia	ND	ND	0.3/0.3	ND	ND
41	S	female	21ys	congenital aniridia	yes/Grade 1b/1b	catarcts, nystagmus	1.0/1.0	45.3/43.3	25.09/24.85
42	F	male	7mos	congenital aniridia	ND	glaucoma, corneal opacity	ND	ND	ND
42-2	F, mother of No.42	female	27ys	congenital aniridia	yes	cataract	1.4/1.5	ND	21.5/-
43	S	male	6ys	congenital aniridia	yes/Grade 3/3	cataracts, glaucoma, nystagmus	1.3/1.0	12/20.2	22.77/23.58
44	S	male	21ys	congenital aniridia	yes/Grade 2/3	cataracts, glaucoma, nystagmus, microcornea in the left eye	1.0/-3.505	22.5/51	22.3/25.6
45	S	male	2ys	congenital aniridia	ND	ND	ND	ND	ND
46	F	female	45ys	congenital aniridia	yes/Grade 4/4	cataracts, left eye lens subluxation	1.3/1.3	14.5/15.6	21.44/22.18
46-2	F, father of No.46	male	80ys	congenital aniridia	ND	cataracts	ND	17/16	ND
47	S	male	1ys	congenital aniridia	yes	ND	ND	ND	ND
48	S	female	9mos	congenital aniridia	ND	ND	ND	ND	ND
49	S	male	4ys	congenital aniridia	ND	ND	ND	ND	ND
50	F	female	25ys	congenital aniridia	ND	cataracts, glaucoma, diabetes	-3.505/2.9	40/14	ND
50-2	F, mother of No.50	female	60ys	congenital aniridia	ND	cataracts, diabetes	ND	ND	ND
51	S	male	14ys	congenital aniridia	yes/Grade 3/3	cataracts, glaucoma, nystagmus	1.4/1.3	40.9/14.8	27.57/24.59

BCVA, best-corrected visual acuity; OD, right eye; OS, left eye; IOP, intraocular pressure; S, sporadic; ys, years; mos, months; FC, fingers counting; BE, before eyes; ND: no data; F, familial; HM, hand movements; NLP, no perception of light

*Her father has aniridia but did not participate in this study. **Structure grading (OD/OS) of foveal hypoplasia based on macular OCT in 15 patients who got OCT examination

**Fig. 1:**
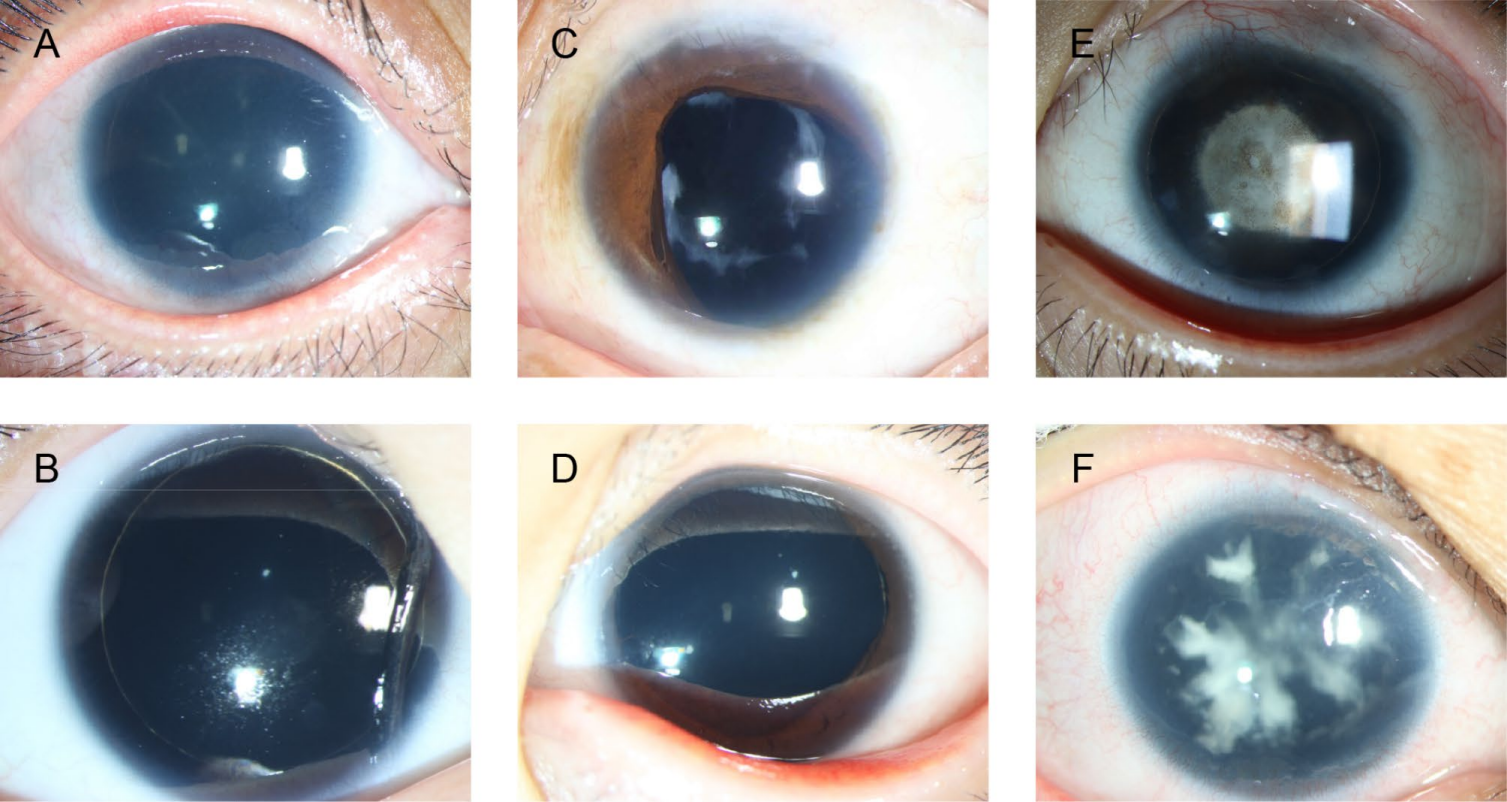
Typical anterior segment photographs of patients with congenital aniridia. **A** and **B** demonstrate complete hypoplasia of the iris in patients No. 4 (**A**) and No. 22 (**B**). C and D show partial iris hypoplasia or iridocoloboma with residual circular peripheral iris in patients No. 4 − 2 (**C**) and No. 30 (**D**). **E** and **F** show characteristic lenticular opacities in patients No. 19 (**E**) and No. 20 (**F**)

**Fig. 2:**
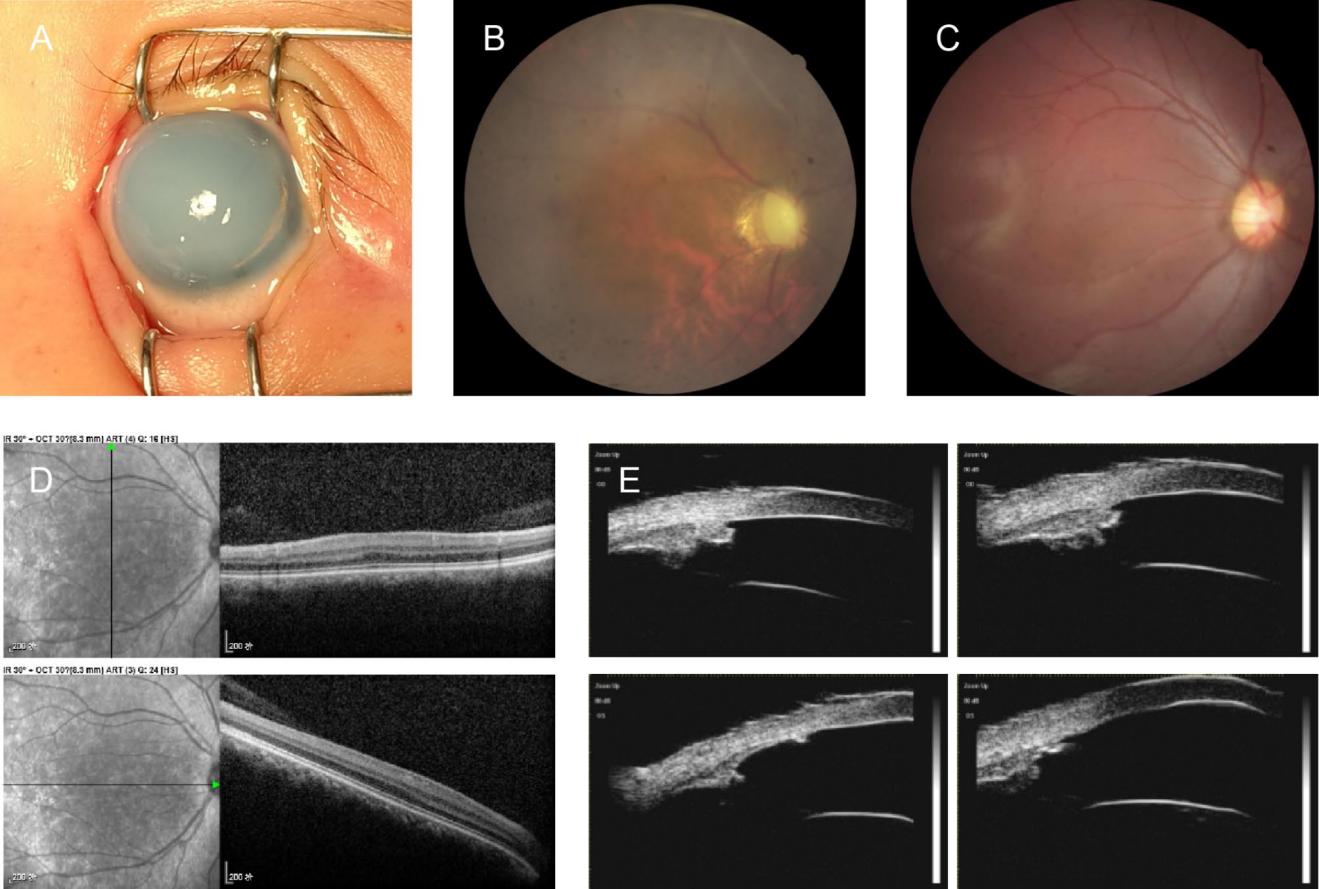
Images of glaucoma, foveal hypoplasia, and congenital aniridia—anterior segment, fundus, OCT, UBM. (**A**) cornea edema and opacity in patient No. 36. (**B**) large cup-disc ratio and pale optic nerve in patient No. 27 − 2. (**C**) no macular reflex halo in patient No. 21. (**D**) flat macular fovea in patient No. 30 − 2. (**E**) only iris stump and unclear scleral process in patient No. 44

Of the 60 patients with congenital aniridia, 58 exhibited complete hypoplasia of the iris (Fig. 1A and B), and two (No. 30 and No. 4 − 2) showed partial iris hypoplasia with a residual circular peripheral iris (Fig. 1C and D). Thirty-four patients (34/60, 56.7%) had binocular cataracts (Fig. 1E and F), and 23 patients (23/60, 38.3%) had glaucoma (Fig. 2A and B). Thirty-five patients, whose fundi were examined using ophthalmoscopy or OCT, presented with foveal hypoplasia (Fig. 2C and D). Seventeen patients underwent UBM examination, which revealed only an iris stump and dysplastic scleral process or even close angles (Fig. 2E). Two children (No. 9 and No. 31) were diagnosed with WAGR syndrome, and one (No. 31) was diagnosed with nephroblastoma through renal ultrasound examination after a positive molecular genetic test. Additionally, one case of microcornea was observed (No. 44), while ectopia lentis was identified in four eyes of three patients (No. 3, No. 24 and No. 46). Furthermore, nystagmus was documented in 20 participants. Intrafamilial phenotypic heterogeneity was observed in the No. 4 family. The patient No. 4 showed total aniridia in both eyes (Fig. 1A), whereas her father (No. 4 − 2) displayed only partial iridocoloboma in both eyes (Fig. 1C). In addition, one family member (No. 30 − 2, the mother of patient No. 30) exhibited no discernible iris abnormalities, yet displayed nystagmus and foveal hypoplasia.

## Genetic test

### Panel-based NGS and variant analysis

Among the 51 families, panel-based NGS revealed 32 mutations in *PAX6* (GenBank NM_000280.5) and one mutation in *BCOR* (GenBank NM_001123385.2) in 36 probands. These mutations included nine deletion/insertion mutations and 24 point mutations with three splicing mutations, as shown in Table 2 and Supplement 1 (a summary of the clinical findings and genetic test results for the 60 patients). All mutations were verified via Sanger sequencing in 36 probands and seven family members with congenital aniridia. In this study, the mutation detection rate of panel-based NGS in congenital aniridia patients was 71.7% (43/60).

**Table 2:** Panel-based NGS revealed mutations involved in *PAX6* gene

No.	Nucleotide variants	Amino acid change	Exon/Domain	ACMG	Reference
1	c.28 C > T	p.Gln10Ter	exon5/PD	P	[17]
2	c.34G > C	p.Gly12Arg	exon5/PD	LP	[36]
3	c.49_61del	p.Asn17Cysfs*10	exon5/PD	LP	this study/
4	c.65_66insTGCC	p.Asp23Alafs*34	exon5/PD	LP	this study/
5	c.94 C > G	p.Leu32Val	exon5/PD	VUS	[5, 6]
6	c.102_103del	p.His34Glnfs*21	exon5/PD	LP	this study/
7	c.140 A > T	p.Gln47Leu	exon5/PD	LP	this study/
8	c.183 C > G	p.Tyr61Ter	exon6/PD	P	[34]
9	c.300G > A	p.Trp100Ter	exon6/PD	P	[41]
10	c.307 C > T	p.Arg103Ter	exon6/PD	P	[13]
11	c.308delG	p.Arg103Glnfs*21	exon6/PD	P	[2]
12	c.356delG	p.Ser119Thrfs*5	exon6/PD	LP	this study/
13	c.357 + 2insATAACA	-	exon6	LP	this study/
14	c.468G > A	p.Trp156Ter	exon7/LNK	P	[4]
15	c.485G > A	p.Trp162Ter	exon7/LNK	P	[46]
16	c.520 C > T	p.Gln174Ter	exon7/LNK	P	[42]
17	c.531 C > A	p.Cys177Ter	exon8/LNK	LP	this study/
18	c.590 C > G	p.Ser197Ter	exon8/LNK	LP	[6]
19	c.607 C > T	p.Arg203Ter	exon8/LNK	P	[29]
20	c.683–2 A > G	-	exon9	LP	this study/
21	c.718 C > T	p.Arg240Ter	exon9/HD	P	[29]
22	c.765G > T	p.Gln255His	exon9/HD	VUS	[3]
23	c.765 + 1G > T	-	exon9	LP	[35]
24	c.781 C > T	p.Arg261Ter	exon10/HD	LP	[15]
25	c.781dupC	p.Arg261Profs*23	exon10/HD	LP	this study/
26	c.802G > T	p.Glu268Ter	exon10/HD	LP	[45]
27	c.808 A > T	p.Lys270Ter	exon10/HD	LP	this study/
28	c.812_813del	p.Leu271Glnfs*12	exon10/PST	P	[30]
29	c.889 C > T	p.Gln297Ter	exon10/PST	LP	[48]
30	c.949 C > T	p.Arg317Ter	exon11/PST	LP	[8]
31	c.1154delC	p.Pro385Glnfs*140	exon12/PST	LP	this study/
32	c.1268 A > T	p.Ter423Leu	exon13/PST	P	[4]

*Abbreviations* ACMG, classification of variants according to American College of Medical Genetics; P, pathogenic; LP, likely pathogenic; VUS, variants of uncertain significance; PD, paired domain; PTC, premature termination codon; PST, proline-threonine-serine-rich domain; LNK, linker region; HD, homeodomain. The sequencing results were compared with published cDNA sequence for *PAX6* (GenBank NM_000280.5). Novel variants found in this study were submitted to ClinVar with accession numbers SCV004242327-SCV004242337(https://www.ncbi.nlm.nih.gov/clinvar/)

**Fig. 3:**
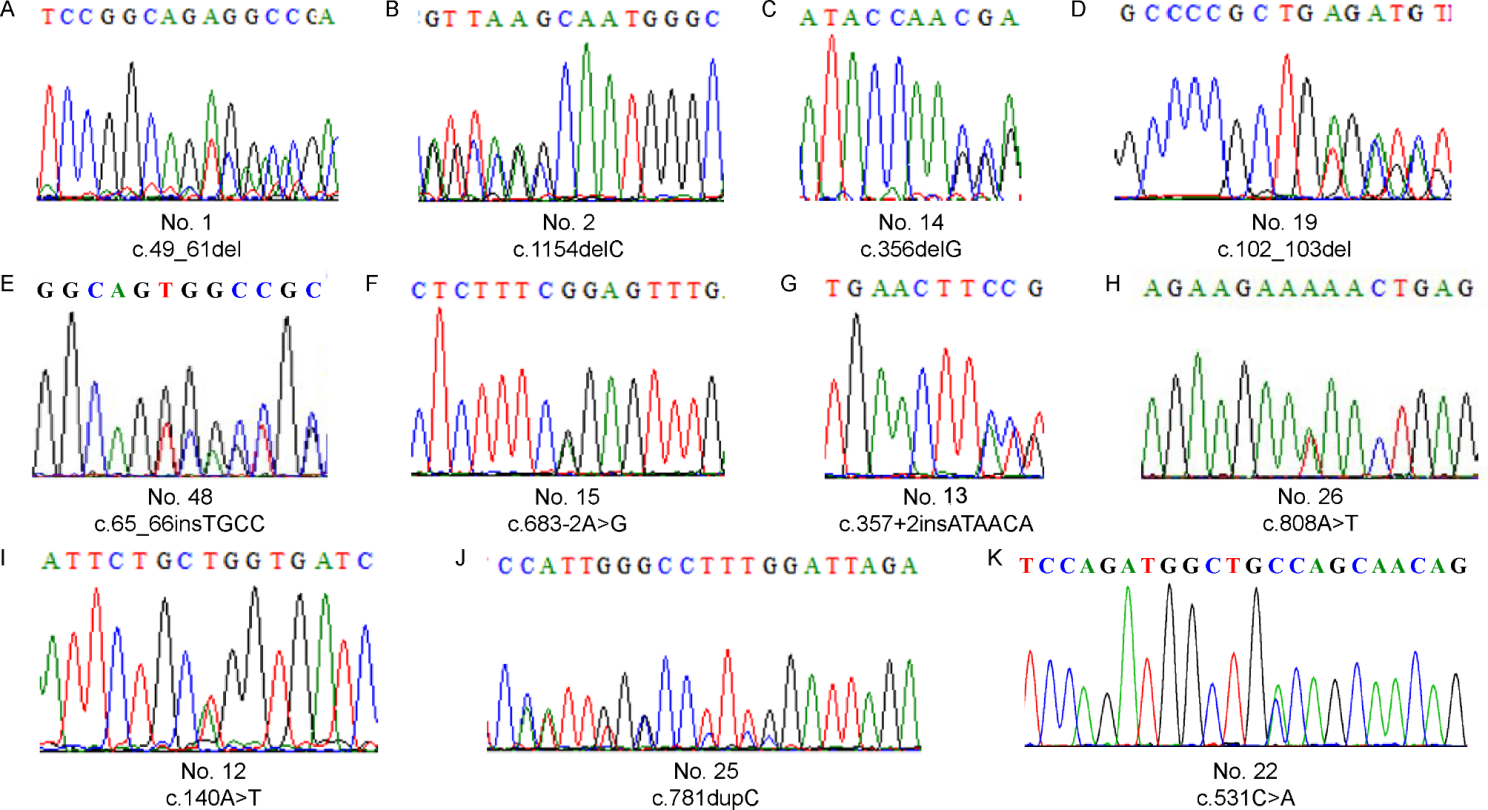
Novel *PAX6* mutations identified in this study. A-K: sequencing chromatograms of c.49_61del, c.1154delC, c.356delG, c.102_103del, c.65_66insTGCC, c.683–2 A > G, c.357 + 2insATAACA, c.808 A > T, c.140 A > T, c.781dupC and c.531 C > A in *PAX6* gene

**Fig. 4:**
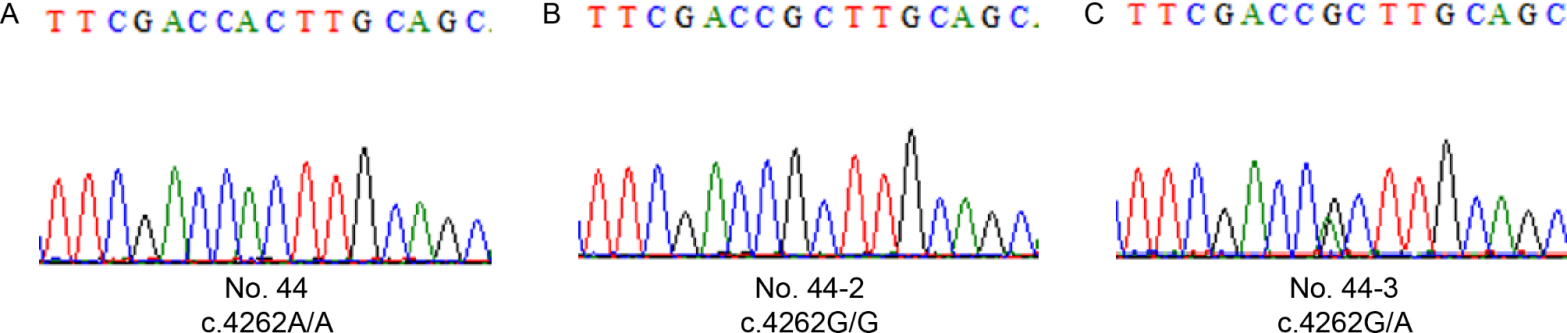
The mutation c.4262G > A in *BCOR* gene identified in this study. **A**-**C**: sequencing chromatograms of No. 44 (proband), No. 44 − 2 (father), and No. 44 − 3 (mother), respectively

Of these 33 mutations (32 in *PAX6* and 1 in *BCOR*), 12 have not been reported in previous studies, including six frameshift mutations (c.49_61del, c.1154delC, c.356delG, c.102_103del, c.781dupC, c.65–66 insTGCC), one missense mutation (c.140 A > T), two splicing mutations (c.357 + 2 insATAACA, c.683–2 A > G), two nonsense mutations (c.531 C > A and c.808 A > T) in *PAX6* (Fig. 3), and one missense mutation (c.4262G > A) in *BCOR* (Fig. 4). Intriguingly, we found that two family members (No. 30 − 2 and No. 44 − 3) carried gene mutations but did not clinically show iris hypoplasia. Patient No. 30 − 2, the mother of proband No. 30, was found to carry c.94 C > G in *PAX6*, which was consistent with her daughter, who presented with iridocoloboma and a residual circular peripheral iris. Patient No. 44 presented with binocular congenital aniridia, cataract, and microcornea in his left eye with white-to-white distance of 12.20 mm in his right eye and 10.80 mm in his left eye, measured with Lenstar LS 900 (HAAG-STREIT DIGNOSTICS, EyeSuite™ Biometry, V2.5.2). He was found to have a hemizygous missense variation c.4262G > A (p.Arg1421His) in *BCOR*, which is located on chromosome Xp11.4. This mutation was inherited from his mother (No. 44 − 3), who had strabismus but no aniridia or microcorneas, and was not detected in his father (No-44-2), who was phenotypically normal.

### MLPA results

**Fig. 5:**
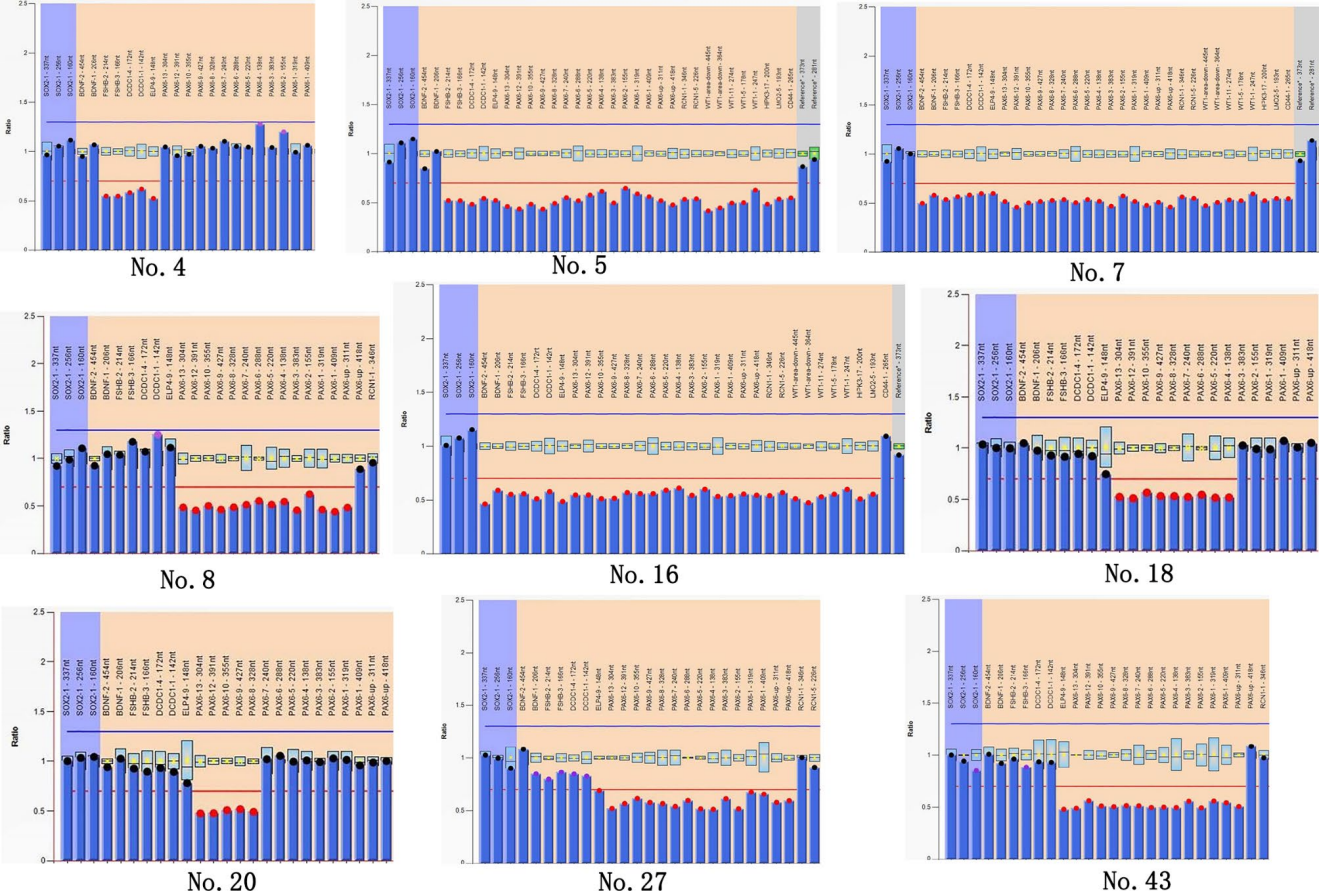
Representative MLPA figures of *PAX6* and adjacent genes. The blue columns with red top dots represent the deleted probes with peak areas below 0.65. The blue columns within grey areas indicate the control probes

**Fig. 6:**
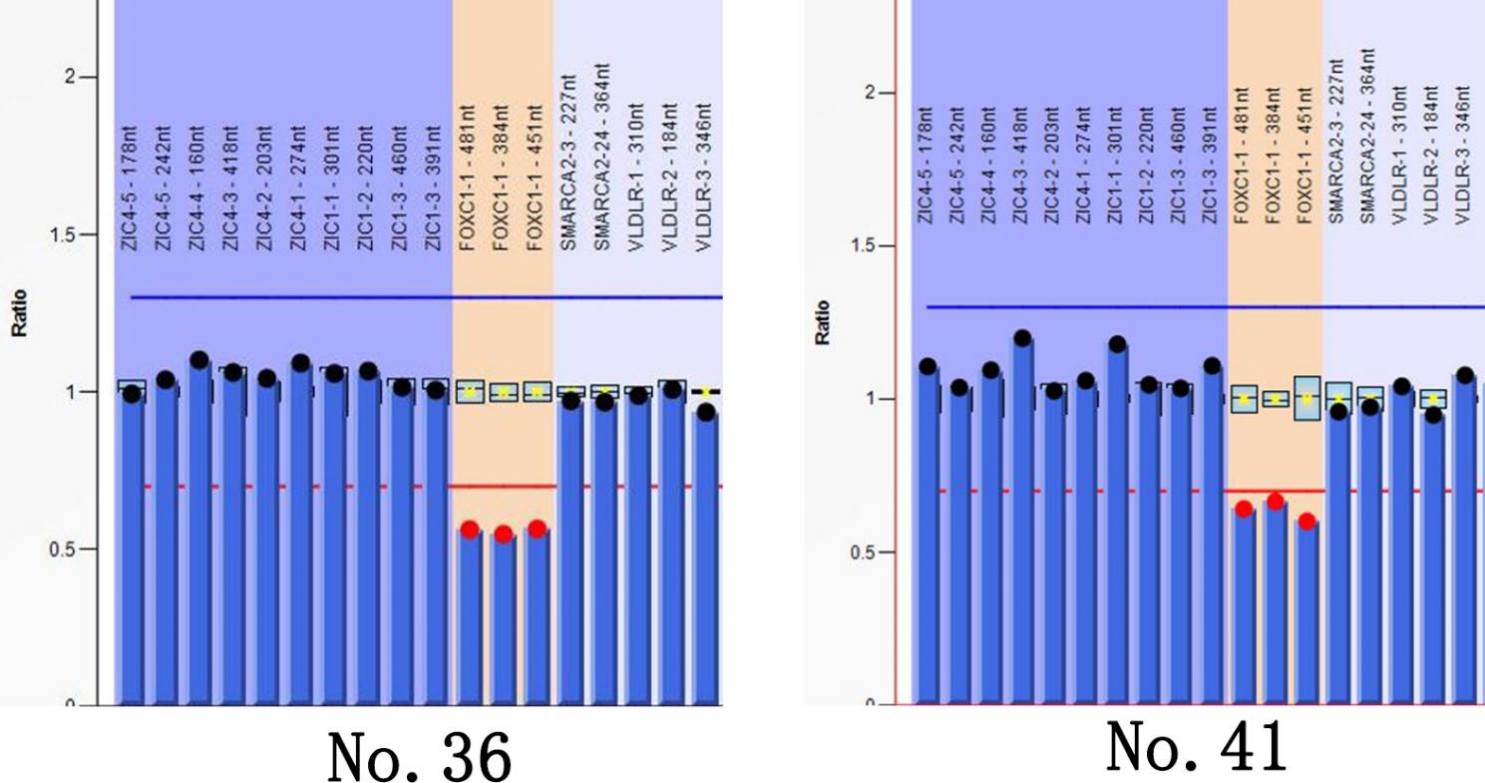
MLPA figures of *FOXC1* deletions. The blue columns with red top dots depict the deleted probes with peak areas below 0.65. The blue columns within grey areas represent the control probes

MLPA was performed on 15 families in whom no gene mutations were detected via NGS. Among them, heterozygous segmental loss of *PAX6* or contiguous genes was observed in 12 families, and heterozygous *FOXC1* (GenBank NM_001453.3) deletions were detected in two families, as shown in Supplement 1. Two out of the 15 probands had positive family histories, and affected family members were found to carry the same deletion as the probands in their families. The MLPA results for individual probands are shown in Figs. 5 and 6. In one patient (No. 39) without intragenic mutations (as detected using NGS), no deletions were found with MLPA for *PAX6* and *FOXC1* either. The overall mutation detection rate in this study for congenital aniridia patients was 98.3% (59/60) when panel-based NGS and MPLA methods were combined.

## Discussion

In this study, we enrolled 51 probands with congenital aniridia and their family members (60 patients). Nine family members were clinically diagnosed with aniridia and related diseases, and one family member presented with nystagmus and foveal hypoplasia. All ocular iris abnormalities were almost completely symmetrical in both eyes. Among the 60 patients, 23 (23/60, 38.3%) were diagnosed with glaucoma at the first visit, similar to the reported 30–67% prevalence of glaucoma in *PAX6*-associated congenital aniridia [27]. However, only 34 patients (34/60, 56.7%) had varying degrees of opaque lenses, lower than the 70–85% reported in other studies [27]. A possible reason for this is the invisible intraocular conditions due to severe corneal edema and opacity in a proportion of glaucoma patients. In two children diagnosed with WAGR syndrome, one child (No. 31) was diagnosed with nephroblastoma via renal ultrasound examination based on the implications of a positive genetic test with a large deletion covering *PAX6* and *WT1*.

Using panel-based NGS and MLPA, intragenic mutations or deletions in *PAX6* were identified in 54 patients, accounting for 90% (54/60) of the total. Among the 60 patients with congenital aniridia, two patients showed only partial iridocoloboma in both eyes (No. 4 − 2 and No. 30). Patient No. 4 (daughter of No. 4 − 2) showed complete aniridia in both eyes, although patient No. 4 and patient No. 4 − 2 carried identical heterozygous deletions covering *FSHB*, *DCDC1*, and *ELP4* adjacent to *PAX6*. Moreover, patient No. 30 presented with iridocoloboma and a residual circular peripheral iris, while her mother (No. 30 − 2) presented with nystagmus, myopia, astigmatism, and binocular macular foveal dysplasia without obvious iris abnormalities, although both carried c.94 C > G in *PAX6*. This suggests that the phenotype of *PAX6*-associated aniridia is variable, even in intrafamilial patients. Phenotypic inconsistencies within families have also been observed in other studies [10, 33, 45]. The specific mechanism underlying this phenotypic variability within the family remains unclear and requires further investigation.

In addition to aniridia, *PAX6* mutations can also cause foveal dysplasia or affect only the fovea without the involvement of the iris, which is a rare medical condition involving the underdevelopment of the macula. For *PAX6*-associated foveal hypoplasia, Gottlob I et al. found a *PAX6* missense mutation (c.227 C > G, p.Pro76Arg) in a large multigenerational white British family with normal irides and foveal hypoplasia [40]. Zhang et al. reported *PAX6* mutations in families with isolated foveal hypoplasia and a full iris; however, careful follow-up visits revealed subtle structural abnormalities in the iris in more than half of the patients with *PAX6* mutations [21]. In our study, two participants from one family showed macular dysplasia; proband No. 30 showed partial iris hypoplasia, but her mother (No. 30 − 2) had a full iris. Both carried the same *PAX6* mutation-p.Leu32Val located in the N-terminal subdomain, which was not consistent with the genotype and phenotype pattern of *PAX6*, as shown in a previous report [28]. This indicates that other factors, such as modified factors (genetic, epigenetic, or environmental), may be involved in the phenotypic differences within families, in addition to the genetic variants themselves [7].

In addition to *PAX6*, aniridia-like phenotypes have been observed in patients with rare variants in *FOXC1* and *PITX2* as well [1, 20, 26]. We also found *FOXC1* deletions in two patients (No. 36 and No. 41) using MLPA. Patient No. 36 presented with complete aniridia and severe corneal edema owing to high IOP. Patient No. 41 presented with complete aniridia, cataracts, and nystagmus. The mutation detection rate of *FOXC1* in this study was 3.3% (2/60), which was slightly lower than the rates of 7–13% reported by other studies [1, 24, 32].

Surprisingly, we detected a *BCOR* mutation in one patient with congenital aniridia and monocular microcornea. These mutations have been identified in patients with microphthalmia syndrome or oculofaciocardiodental syndrome, which have overlapping ocular phenotypes but different X-linked inheritance patterns [18, 31]. Our patient carrying *BCOR* c.4262G > A, who presented with congenital aniridia and monocular microcornea, had a phenotypic overlap with a patient carrying *BCOR* c.4870 C > T, as reported in a previous study [38]. The full-length *BCOR* protein functions as a co-repressor of BCL6, a POZ/zinc finger transcriptional repressor [19]. Additionally, knockdown of the zebrafish ortholog of *BCOR* causes developmental perturbations of the eye and other organs that mimic human symptoms, confirming that *BCOR* interacts with transcriptional partners other than BCL-6 and is a key transcriptional regulator during early embryogenesis [31]. The contribution of *BCOR* to eye development requires further investigation.

In this cohort study involving 60 patients, genetic abnormalities were identified in 59 patients. Among these aniridia cases, deletions in *PAX6* and its adjacent genes were identified in 12 patients, and small intragenic *PAX6* variants were detected in 42 patients including 11 novel and 21 previously reported mutations. Deletions of genes adjacent to *PAX6* were identified in two patients, *FOXC1* deletions in two patients, and an intragenic *BCOR* mutation in one patient. In this study, the mutation detection rate of panel-based NGS in classic congenital aniridia patients was 71.7% (43/60). When combined with MLPA, the mutation detection rate increased to 98.3% (59/60), demonstrating that MLPA substantially enhanced the molecular diagnosis of aniridia. The detection rate of 98.3% was higher than that (55%) reported in a Chinese cohort of 38 patients [47] and that (96.9%) reported in 95 Chinese aniridia patients using Sanger sequencing and MLPA for only *PAX6* [46]. In our study, we employed panel-based NGS, including exons of 289 genes involved in ocular anterior segment dysgenesis, and MLPA for *PAX6* and *FOXC1*, which significantly improved the detection rate.

However, the present study enrolled a relatively small number of patients and did not include atypical aniridia cases; clinical data were incomplete in some patients, and some family members with clinical phenotypes did not participate in this study. In the future, the target population should be expanded to include patients with congenital iris hypoplasia. Ultimately, novel, effective treatments or gene therapies for congenital aniridia should be explored and implemented clinically.

## Conclusions

In this cohort study of 60 patients, we identified genetic abnormalities in 59 patients with congenital aniridia using panel-based NGS and MLPA of *PAX6* and *FOXC1*. The total detection rate was 98.3%, which was higher than that reported previously. Panel-based NGS combined with MLPA significantly increases the detection rate of gene mutations in patients with congenital aniridia. This study confirmed that variations in *PAX6* and its adjacent regions were the predominant causes of congenital aniridia. In addition, *FOXC1* is another key gene associated with congenital aniridia. This study highlights the importance of clinical implementation of targeted NGS and MLPA as diagnostic strategies for aniridia and aniridia-like disorders.

## Electronic supplementary material

Below is the link to the electronic supplementary material.

Supplementary Material 1

Supplementary Material 2

## Data Availability

All data generated or analyzed during this study are included in this published article and its supplementary information files.

## Abbreviations

NGS: Next-generation sequencing
MLPA: Multiplex ligation probe amplification
WAGR: Wilms’ tumor-aniridia-genital anomalies-retardation syndrome
EENT Hospital: The Eye, Ear, Nose, and Throat Hospital
IOP: Intraocular pressure measurement
OCT: Optical coherence tomography
BCVA: Best-corrected visual acuity
GATK: Genome Analysis Toolkit version 3.7
